# Association of intellectual disability with overall and type-specific cardiovascular diseases: a population-based cohort study in Denmark

**DOI:** 10.1186/s12916-023-02747-4

**Published:** 2023-02-06

**Authors:** Hui Wang, Priscilla Ming Yi Lee, Jun Zhang, Katrine Svendsen, Fei Li, Jiong Li

**Affiliations:** 1 MOE-Shanghai Key Laboratory of Children’s Environmental Health, Xin Hua Hospital Affiliated to Shanghai Jiao Tong University School of Medicine, Shanghai, China; 2 School of Public Health, Shanghai Jiao Tong University School of Medicine, Shanghai, China; 3 Department of Clinical Medicine-Department of Clinical Epidemiology, Aarhus University, Olof Palmes Alle 43-45, 8200 Aarhus N, Denmark; 4 Research Unit for General Practice, Aarhus University, Aarhus, Denmark; 5 State Key Laboratory of Reproductive Medicine, Nanjing Medical University, Nanjing, China; 6 Department of Epidemiology, School of Public Health, Nanjing Medical University, Nanjing, China

**Keywords:** Intellectual disability, Mental disorders, Cardiovascular disorder, Cohort study, Epidemiology

## Abstract

**Background:**

Individuals with mental health problems have been shown to have an increased risk of cardiovascular disorder (CVD), but little is known about the risk of early-onset CVD among those with intellectual disability. We aimed to investigate the association between intellectual disability and subsequent CVD, taking into consideration the severity of intellectual disability and neurodevelopmental and neurologic comorbidity.

**Methods:**

This population-based cohort study used individual-level linked data from Danish national health registries. Participants were all live-born singletons born in Denmark during 1978–2016 (*n* = 2,288,393). Follow-up began from birth and continued until the onset of CVD, death, emigration, or December 31, 2018, whichever came first. Clinical diagnosis of any CVD or type-specific CVDs was identified in the Danish National Patient Register. Time-varying Cox regression analyses were used to estimate the hazard ratio (HR) of intellectual disability associated with overall and type-specific CVDs.

**Results:**

A total of 11,954 individuals received a diagnosis of intellectual disability (7434 males and 4520 females). During a median follow-up time of 18.5 years (interquartile range, 18.1 years), 652 individuals with intellectual disability (5.5%) received a diagnosis of CVD (incidence rate, 2.4 per 1000 person-years), compared with 78,088 (3.4%) CVD cases in individuals without intellectual disability (incidence rate, 1.9 per 1000 person-years), corresponding to a HR of 1.24 (95% CI, 1.15–1.34). Increased risks of CVD were similar in both childhood (HR, 1.24; 95% CI, 1.08–1.43) and early adulthood (HR, 1.25; 95% CI, 1.14–1.38). For type-specific CVDs, intellectual disability was significantly associated with cerebrovascular disease (HR, 2.50; 95% CI, 2.02–3.10), stroke (HR, 2.20; 95% CI, 1.69–2.86), heart failure (HR, 3.56; 95% CI, 2.37–5.35), hypertensive disease (HR, 1.30; 95% CI, 1.22–1.39), and deep vein thrombosis (HR, 2.10; 95% CI, 1.60–2.75). Stratified HRs of overall CVD were 1.14 (95% CI, 1.01–1.30) for borderline/mild intellectual disability, 1.25 (95% CI, 1.01–1.54) for moderate intellectual disability, and 1.91 (95% CI, 1.47–2.48) for severe/profound intellectual disability. After the exclusion of individuals with neurodevelopmental and neurologic comorbidity, intellectual disability remained significantly associated with increased risks of CVD.

**Conclusions:**

Individuals with intellectual disability had increased risks of early-onset CVD, in particular, for cerebrovascular disease, stroke, heart failure, and deep vein thrombosis, and the risks also increased with the severity of intellectual disability. Our findings highlight the awareness of increased risks of CVD in intellectual disability patients.

**Supplementary Information:**

The online version contains supplementary material available at 10.1186/s12916-023-02747-4.

## Background

Cardiovascular disease (CVD) represents a major public health concern [1] due to its high prevalence, poor prognosis, substantial personal burden, and societal costs [2]. Previous studies have demonstrated a broad range of risk factors for CVD, including metabolic syndrome and unhealthy lifestyle [3, 4]. Despite significant advances in its treatment and prevention, CVD is still the leading cause of morbidity and mortality worldwide, accounting for one-third of all deaths in 2019 [5]. The etiology of CVD is multifactorial, which could not be explained by conventional risk factors [3, 4], highlighting the importance to identify unknown risk factors for better disease management and prevention [6, 7].

There is now an increasing awareness of the role of neurodevelopmental disorders in the development of CVD [8–10]. Intellectual disability is characterized by global deficits in cognitive functioning and adaptive behaviors [11]. The lifetime prevalence of intellectual disability is approximately 1% in the general population [11, 12]. Previous studies have indicated that intellectual disability has been associated with increased risks of adverse health outcomes including hyperlipidemia, diabetes, and obesity that are known risk factors for CVD [13–15]. However, empirical evidence on the association between intellectual disability and CVD remains scarce [9]. Only a few studies have had the statistical power to clarify the overall and type-specific CVD risks associated with intellectual disability, or considered the severity of intellectual disability [15, 16]. Also, intellectual disability tends to be comorbid with other neurodevelopmental disorders [17, 18]. For example, individuals with intellectual disability are significantly more likely to be diagnosed with autism spectrum disorders (ASD) and epilepsy [19, 20]. Improved understanding of the potential contributions by neurodevelopmental and neurologic comorbidity for the associations between intellectual disability and CVD could substantially facilitate surveillance and more targeted prevention strategies and interventions.

Compared to the general population, people with intellectual disability suffer from more health problems and have inequalities such as worse access to health care, premature mortality, and socioeconomic disadvantages [21, 22]. Considering barriers in accessing health care and the vulnerability of individuals with intellectual disability, understanding the needs of people with intellectual disability and awareness of the implications of the disorder is important to health care planning. This study will address an important research gap about the CVD outcomes associated with intellectual disability.

To estimate the risk of overall and type-specific CVD in individuals with intellectual disability, a study with a large sample size and long follow-up is needed. This nationwide Danish cohort study with a follow-up of up to 40 years would provide such an opportunity [23, 24]. Specifically, in this study, we aimed to (1) explore the overall and type-specific CVD risk in individuals with intellectual disability, taking into account the severity of intellectual disability, and whether the associations were consistent across childhood and early adulthood and (2) examine whether the comorbid neurodevelopmental and neurologic disorders contribute to the risk of CVD. If the association between intellectual disability and subsequent CVD is confirmed in this study, intellectual disability may be considered as a novel risk factor for CVD, and individuals with intellectual disability could be a target population for the primary prevention of CVD in young adults.

## Methods

### Design and population

We conducted a nationwide cohort study using data from national registers in Denmark [23–27] whose descriptions are provided in Additional file 1: Table S1. In Denmark, all live births have a unique personal identification number that permits an accurate linkage of individual-level data [25]. We identified all singleton live births in Denmark from 1978 to 2016 (*n* = 2,330,627). After excluding (1) 557 who had extreme gestational age (< 154 or > 315 days), (2) 1001 without information on sex, (3) 7274 with congenital malformations of the nervous system (the *International Classification of Disease, Eighth Revision* [*ICD-8*] codes 740–743; *Tenth Revision* [*ICD-10*] codes Q00–Q07), (4) 8025 with chromosomal abnormalities (*ICD-8* codes 7589, 759; *ICD-10* codes Q90–Q99), (5) 23,186 with congenital heart disease (*ICD-8* codes 740–759; *ICD-10* codes Q20–Q26), and (6) 2191 diagnosed with CVD before a diagnosis of intellectual disability, the final cohort included 2,288,393 individuals in Denmark (as shown in Additional file 1: Fig. S1). We followed them from birth until the date of the first diagnosis of any or a specific type of CVD event, emigration, death, or end of follow-up (December 31, 2018), whichever came first.

### Assessment of exposures

Information on intellectual disability was obtained from the combination of the Danish National Patient Register (DNPR) and the Danish Psychiatric Central Research Register (DPCRR) in Denmark, using the *ICD* codes [25, 26]. The DNPR contains hospital discharge diagnoses from 1977 and outpatient and emergency diagnoses since 1995 [25]. The DPCRR contains information on all individuals with psychiatric disorders treated in secondary care since 1970, and outpatient and emergency department contact were also included since 1995 [26]. Denmark has used the *ICD-8* up to 1993 and *ICD-10* since 1994. Intellectual disability was identified using *ICD-8* codes 310–315 and *ICD-10* codes F70–F79. Intellectual disability was further categorized into four groups according to the severity of the disorder: borderline/mild intellectual disability (*ICD-8* codes 310–311; *ICD-10* code F70), moderate intellectual disability (*ICD-8* code 312; *ICD-10* code F71), severe/profound intellectual disability (*ICD-8* codes 313–314; *ICD-10* codes F72–F73), and others or unspecified intellectual disability (*ICD-8* code 315; *ICD-10* codes F78–F79) [28, 29].

The diagnoses of neurodevelopmental and neurologic comorbidity were also obtained from DNPR and DPCRR [25, 26] and included (1) attention-deficit/hyperactivity disorder (ADHD), (2) autism spectrum disorder (ASD), (3) epilepsy, (4) cerebral palsy, and (5) intracranial tumors, head trauma, and intracranial infection (specific codes are provided in Additional file 1: Table S2).

### Ascertainment of CVD

Information on CVD was obtained from the DNPR [25, 30, 31]. Our primary outcome was the first diagnosis of any CVD using *ICD* codes (*ICD-8* codes 390–444.1, 444.3–458, 782.4; *ICD-10* codes I00–I99). With the large study sample and a long follow-up, we were able to categorize CVD into the following specific diagnostic groups: (1) ischemic heart disease, (2) cerebrovascular disease, (3) stroke, (4) heart failure, (5) atrial fibrillation, (6) hypertensive disease, and (7) deep vein thrombosis (specific codes are provided in Additional file 1: Table S3). When investigating the type-specific CVD, we defined the date of onset as the first day of each specific diagnosis, irrespective of other CVD diagnoses, if existed.

### Covariates

Based on previous research [8, 28], the following variables were considered as potential confounders: sex (male, female), calendar period of birth (a 5-year interval during 1978–2016), parity (1, 2, ≥ 3), maternal age at birth (≤ 25, 26–30, 31–35, ≥ 36 years), maternal country of origin (Denmark, other countries), maternal education level (0–9, 10–14, ≥ 15 years), maternal cohabitation status at birth (yes, no), and maternal psychiatric disorder before the childbirth (yes, no).

### Statistical analysis

Cox proportional hazards regression model with the individual’s age as the time scale was used to estimate the hazard ratio (HR) with 95% confidence intervals (CI) for the association of intellectual disability with the risk of overall and specific CVD, taking the severity of intellectual disability into account. In addition to underlying attained age, model 1 adjusted for sex and calendar year of birth, and model 2 additionally adjusted for parity, maternal age at birth, maternal education level, maternal cohabitation, and maternal psychiatric disorders before childbirth. We used the robust sandwich estimator for standard errors to account for the clustering of individuals within nuclear families bound by the same biological mother [32]. In these models, we treated intellectual disability as a time-varying exposure, where individuals were assumed to be unexposed before the date of intellectual disability diagnosis and exposed after the diagnosis. Kaplan–Meier curves were used to illustrate the probability of CVD diagnosis in exposed and unexposed groups.

While investigating overall CVD risk, we modeled the interaction between intellectual disability and different age bands (1–17 years as childhood and 18–39 years as adulthood) to test whether the associations differed in children and adults. As there are a limited number of specific CVD events in this relatively young population, we did not apply the same analytical strategy to type-specific CVDs. We also stratified the analysis by sex to test whether the results were stable across the sexes, because male and female individuals with intellectual disability may present with different patterns of neurodevelopmental disorders comorbidity [33].

We performed some sensitivity analyses. First, we tested whether the associations varied by preterm birth and maternal psychiatric disorders. Second, given the change in *ICD* revisions (*ICD*-10 was adopted in 1994 in Denmark) and the offspring neurodevelopmental disorders identification strategy (all outpatient diagnoses were available since 1995 in Denmark), we restricted the analysis to offspring born after 1995. Third, to check whether the overall estimates of the association between intellectual disability and risk of CVD will be affected, we included individuals diagnosed with chromosomal abnormalities or congenital heart disease. All tests were two-sided and were considered statistically significant at *p* < 0.05. All statistical analyses were performed using Stata, version 15.1 (StataCorp).

## Results

### Descriptive statistics

The study cohort included 2,288,393 individuals, with a median length of follow-up of 18.5 years (interquartile range, 18.1 years). We identified 11,954 patients (7434 males and 4520 females) with a diagnosis of intellectual disability (5164 [43.2%] with borderline/mild intellectual disability, 1688 [14.1%] with moderate intellectual disability, 769 [6.4%] with severe/profound intellectual disability, and 4333 [36.3%] with unclassified intellectual disability). The median age at intellectual disability diagnosis was 7.8 years (interquartile range, 3.7–14.9 years). Compared with individuals without intellectual disability, individuals with intellectual disability were more likely to be male, born preterm, and had lower Apgar scores at 5 min. Mothers of individuals with intellectual disability were more likely to have lower educational attainment, younger age, and a history of psychiatric disorders before pregnancy (Table 1).

**Table 1:** Baseline characteristics of all individuals with intellectual disability and individuals without intellectual disability born in Denmark (1978–2016)

Characteristics	Individuals with ID, *n* (%)	Individuals without ID, *n* (%)
Sex		
Boys	7434 (62.2)	1,167,151 (51.3)
Girls	4520 (37.8)	1,109,288 (48.7)
Preterm birth (< 37 gestational weeks)		
Yes	1197 (10.0)	97,620 (4.3)
No	10,315 (86.3)	2,098,080 (92.2)
Missing	442 (3.7)	80,739 (3.5)
Apgar score at 5 min		
10	10,138 (84.8)	2,089,318 (91.8)
≤ 9	1555 (13.0)	149,585 (6.6)
Missing	261 (2.2)	37,536 (1.6)
Parity		
1	5000 (41.8)	1,019,903 (44.8)
2	4148 (34.7)	846,076 (37.2)
≥ 3	2806 (23.5)	410,460 (18.0)
Maternal education level (years)		
≤ 9	6272 (52.5)	597,386 (26.2)
10–15	4005 (33.5)	974,963 (42.8)
≥ 16	1395 (11.6)	648,258 (28.5)
Missing	282 (2.4)	55,832 (2.5)
Maternal age (years)		
≤ 24	3685 (30.8)	460,732 (20.2)
25–29	4062 (34.0)	832,359 (36.6)
30–34	2865 (24.0)	677,836 (29.8)
≥ 35	1342 (11.2)	305,512 (13.4)
Maternal original		
Not born in Denmark	1497 (12.5)	260,364 (11.4)
Born in Denmark	10,441 (87.4)	2,009,997 (88.3)
Missing	16 (0.1)	6078 (0.3)
Maternal psychiatric disorders		
Yes	1250 (10.5)	160,848 (7.1)
No	10,686 (89.5)	2,112,473 (92.9)
Age of the participants		
≤ 10	564,468 (25.8)	678 (5.9)
11–19	597,087 (27.2)	3390 (29.7)
20–29	585,249 (26.7)	4591 (40.2)
≥ 30	444,187 (20.3)	2759 (24.2)

*ID* Intellectual disability

### Risk of overall and type-specific CVD

**Fig. 1:**
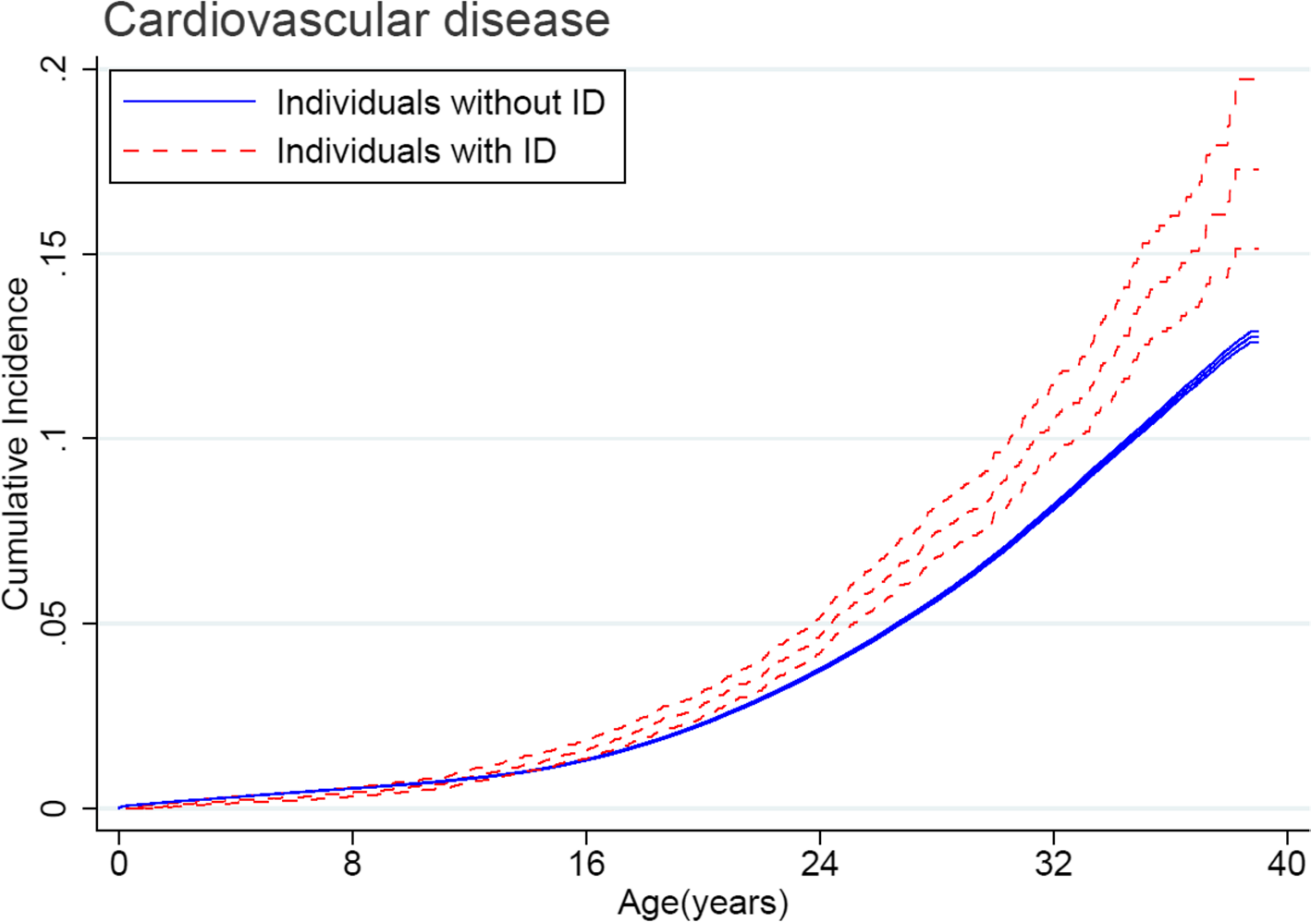
Cumulative incidence of overall cardiovascular disease among individuals with intellectual disability versus individuals without intellectual disability

During the study period, 652 individuals with intellectual disability (5.5%) received a diagnosis of any CVD (incidence rate, 2.4 per 1000 person-years), compared with 78,088 (3.4%) in individuals without intellectual disability (incidence rate, 1.9 per 1000 person-years). Individuals with intellectual disability had a higher risk of developing CVD (Fig. 1). The estimates of cumulative incidences of CVD by 39 years of age were 17.3% (95% CI, 15.1–19.7%) for individuals with intellectual disability and 12.7% (95% CI, 12.6–12.9%) for individuals without intellectual disability. Overall, at the whole population level, intellectual disability was associated with a significantly increased risk of overall CVD (HR, 1.24; 95% CI, 1.15–1.34). The adjusted HR in childhood (1.24; 95% CI, 1.08–1.43) was similar to the HR in early adulthood (1.25; 95% CI, 1.14–1.38) (Table 2). Specifically, intellectual disability was associated with increased risks of cerebrovascular disease (HR, 2.50; 95% CI, 2.02–3.10), stroke (HR, 2.20; 95% CI, 1.69–2.86), heart failure (HR, 3.56; 95% CI, 2.37–5.35), hypertensive disease (HR, 1.30; 95% CI, 1.22–1.39), and deep vein thrombosis (HR, 2.10; 95% CI, 1.60–2.75). Analyses stratified by sex indicated that the HRs of overall CVD did not vary (male, 1.26; 95% CI, 1.13–1.39; female, 1.24; 95% CI, 1.10–1.39; the *p*-value for interaction = 0.92). Nevertheless, we observed sex differences for some specific CVD. The risk of ischemic heart disease was higher in females than in males (HR, 2.02; 95% CI, 1.19–3.43 vs HR, 0.92; 95% CI, 0.55–1.53), as were the risks of heart failure (HR, 5.43; 95% CI, 3.05–9.66 vs HR, 2.65; 95% CI, 1.49–4.70) and deep vein thrombosis (HR, 2.50; 95% CI, 1.83–3.42 vs HR, 1.44; 95% CI, 0.85–2.44 vs) (Table 3).

**Table 2:** HRs for the associations between intellectual disability and overall CVD in childhood and adulthood among all individuals born in Denmark between 1978 and 2016

Group	No. of CVD	Follow-up (person-years)	Rate*	Model 1, HR (95% CI)	Model 2, HR (95% CI)
Overall					
Non-ID	78,088	42,269,874	1.85	1 [reference]	1 [reference]
ID	652	271,731	2.40	1.30 (1.20 to 1.40)	1.24 (1.15 to 1.34)
Childhood					
Non-ID	25,137	29,741,217	0.85	1 [reference]	1 [reference]
ID	195	187,350	1.04	1.25 (1.09 to 1.44)	1.24 (1.08 to 1.43)
Adulthood					
Non-ID	52,951	12,528,657	4.23	1 [reference]	1 [reference]
ID	457	84,381	5.42	1.34 (1.22 to 1.47)	1.25 (1.14 to 1.38)

Model 1 adjusted for sex and calendar year; model 2 additionally adjusted for parity, maternal age, maternal education, maternal country of origin, maternal psychiatric disorders, and cardiovascular disorders before childbirth

*HR* Hazard ratio, *CI* Confidence interval, *CVD* Cardiovascular disease

^*^Per 1000 person-years

**Table 3:** HRs for the associations between intellectual disability (ID) and overall and type-specific CVD among all individuals born in Denmark between 1978 and 2016

	Individuals without ID		Individuals with ID		Model 1, HR (95% CI)	Model 2, HR (95% CI)
	No. of CVD	Rate^*^	No. of CVD	Rate^*^		
**All**						
Type-specific CVD						
Ischemic heart disease	3047	0.07	31	0.11	1.46 (1.03 to 2.08)	1.26 (0.87 to 1.81)
Cerebrovascular disease	5065	0.12	88	0.31	2.65 (2.15 to 3.27)	2.50 (2.02 to 3.10)
Stroke	3821	0.09	59	0.21	2.34 (1.81 to 3.03)	2.20 (1.69 to 2.86)
Heart failure	974	0.02	25	0.09	3.81 (2.56 to 5.66)	3.56 (2.37 to 5.35)
Atrial fibrillation	2013	0.05	19	0.07	1.33 (0.84 to 2.09)	1.26 (0.79 to 2.01)
Hypertensive disease	122 714	2.92	982	3.61	1.32 (1.24 to 1.41)	1.30 (1.22 to 1.39)
Deep vein thrombosis	3898	0.09	57	0.20	2.30 (1.77 to 2.98)	2.10 (1.60 to 2.75)
**Male**						
Overall CVD	37 339	1.72	374	2.25	1.30 (1.18 to 1.44)	1.26 (1.13 to 1.39)
Type-specific CVD						
Ischemic heart disease	1925	0.09	15	0.09	1.00 (0.60 to 1.66)	0.92 (0.55 to 1.53)
Cerebrovascular disease	2585	0.12	55	0.32	2.77 (2.12 to 3.62)	2.59 (1.98 to 3.40)
Stroke	1990	0.09	34	0.20	2.22 (1.58 to 3.11)	2.06 (1.46 to 2.90)
Heart failure	576	0.03	13	0.07	2.94 (1.70 to 5.09)	2.65 (1.49 to 4.70)
Atrial fibrillation	1280	0.06	14	0.08	1.39 (0.82 to 2.35)	1.30 (0.75 to 2.24)
Hypertensive disease	61,201	2.84	544	3.27	1.27 (1.17 to 1.38)	1.25 (1.15 to 1.36)
Deep vein thrombosis	1186	0.05	15	0.09	1.60 (0.96 to 2.66)	1.44 (0.85 to 2.44)
**Female**						
Overall CVD	40 749	1.98	278	2.64	1.29 (1.14 to 1.45)	1.24 (1.10 to 1.39)
Type-specific CVD						
Ischemic heart disease	1122	0.05	16	0.14	2.57 (1.57 to 4.21)	2.02 (1.19 to 3.43)
Cerebrovascular disease	2480	0.12	33	0.30	2.46 (1.75 to 3.47)	2.34 (1.65 to 3.32)
Stroke	1831	0.09	25	0.23	2.53 (1.71 to 3.76)	2.40 (1.60 to 3.60)
Heart failure	398	0.02	12	0.11	5.60 (3.15 to 9.94)	5.43 (3.05 to 9.66)
Atrial fibrillation	733	0.04	5	0.04	1.20 (0.50 to 2.89)	1.20 (0.50 to 2.89)
Hypertensive disease	61,513	2.99	438	4.16	1.40 (1.28 to 1.54)	1.38 (1.25 to 1.51)
Deep vein thrombosis	2712	0.13	42	0.38	2.72 (2.01 to 3.69)	2.50 (1.83 to 3.42)

Model 1 adjusted for sex and calendar year; model 2 additionally adjusted for parity, maternal age, maternal education, maternal country of origin, maternal psychiatric disorders, and cardiovascular disorders before childbirth

*HR* Hazard ratio, *CI* Confidence interval, *CVD* Cardiovascular disease

^*^Per 1000 person-years

### Risk of overall and type-specific CVD across the severity of intellectual disability

**Fig. 2:**
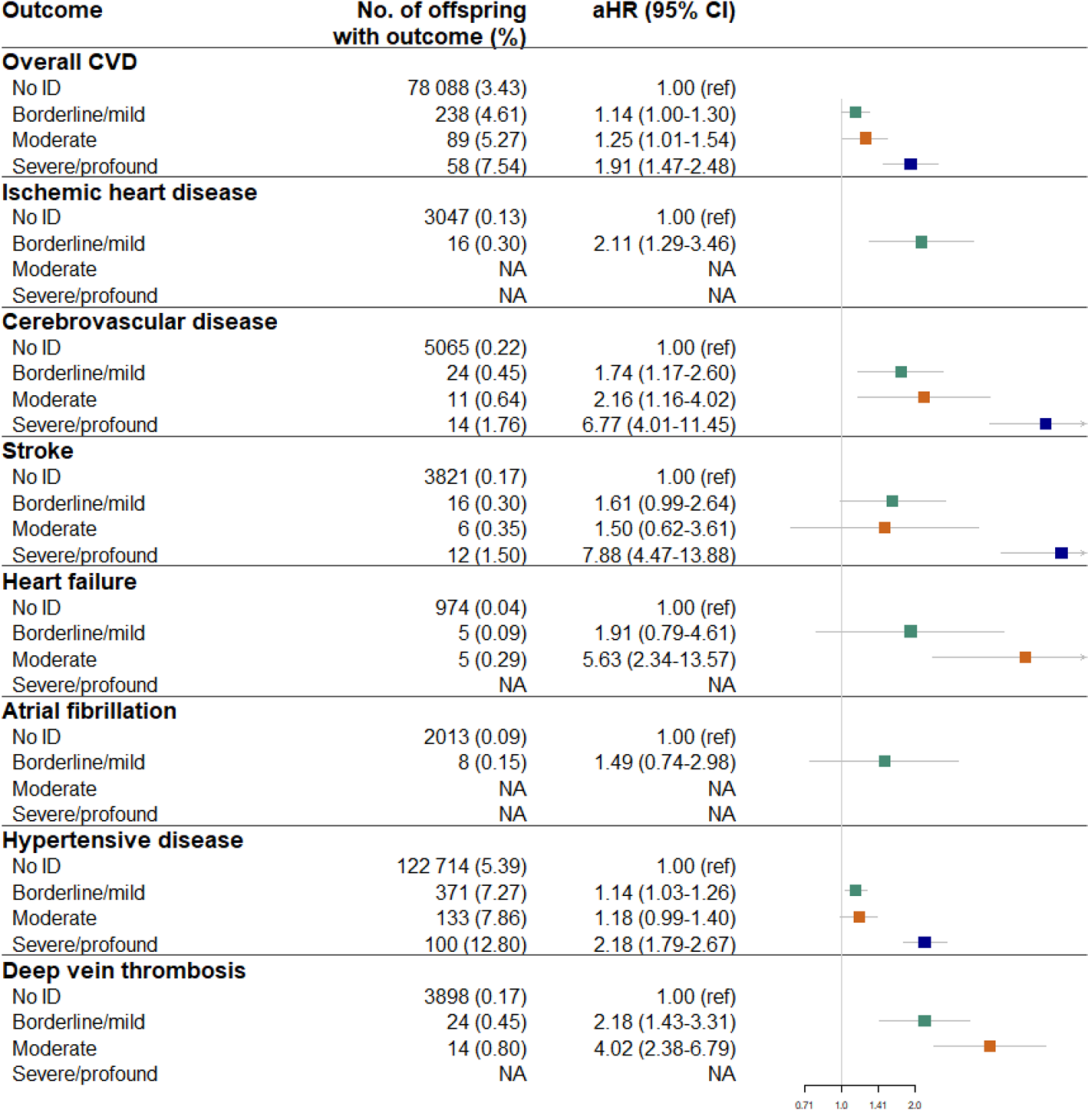
Association between severity of intellectual disability and risk of overall and type-specific cardiovascular disease. Hazard ratios were estimated using Cox proportional hazards regression model, adjusting for sex, calendar year of birth, parity, maternal age at birth, maternal education level, maternal cohabitation, and maternal psychiatric disorders before childbirth. aHR, adjusted hazard ratio; NA, not applicable

When exploring the risk across the severity of intellectual disability, we found that the risk of being diagnosed with CVD increased with the severity of intellectual disability. For overall CVD, the HR was 1.14 (95% CI, 1.01–1.30) for individuals with borderline/mild intellectual disability, 1.25 (95% CI, 1.01–1.54) for moderate intellectual disability, and 1.91 (95% CI, 1.47–2.48) for severe/profound intellectual disability. A similar pattern of results was observed for other specific CVD subtypes. For example, the HRs of cerebrovascular disease for individuals with borderline/mild intellectual disability, moderate intellectual disability, or severe/profound intellectual disability were 1.80 (95% CI, 1.21–2.69), 2.24 (95% CI, 1.20–4.17), and 6.96 (95% CI, 4.12–11.76), respectively (Fig. 2).

### Risk of overall and type-specific CVD after excluding comorbid neurodevelopmental and neurologic disorders

Among individuals with intellectual disability, 2269 (19.0%) had a comorbid diagnosis of ADHD, 2128 (17.8%) had ASD, 1322 (11.1%) had cerebral palsy, 734 (6.1%) had epilepsy, and 407 (3.4%) had intracranial tumors, head trauma, or intracranial infection. We observed that the excess CVD risk was only slightly reduced when excluding these comorbid neurodevelopmental and neurologic disorders (Table 4).

**Table 4:** HRs for the associations between intellectual disability and overall and type-specific CVD among all individuals born in Denmark between 1978 and 2016

	HR (95% CI)^a^				
	Excluding ADHD	Excluding ASD	Excluding epilepsy	Excluding cerebral palsy	Excluding i ntracranial tumors^b^
Overall CVD	1.24 (1.14 to 1.35)	1.23 (1.13 to 1.33)	1.18 (1.09 to 1.28)	1.12 (1.02 to 1.22)	1.19 (1.10 to 1.29)
Ischemic heart disease	1.12 (0.74 to 1.68)	1.21 (0.82 to 1.78)	1.17 (0.79 to 1.74)	1.17 (0.79 to 1.73)	1.26 (0.87 to 1.81)
Cerebrovascular disease	2.63 (2.10 to 3.29)	2.43 (1.93 to 3.05)	2.02 (1.58 to 2.59)	1.77 (1.36 to 2.31)	1.82 (1.41 to 2.33)
Stroke	2.23 (1.68 to 2.94)	2.10 (1.58 to 2.78)	1.78 (1.31 to 2.39)	1.59 (1.15 to 2.19)	1.59 (1.16 to 2.16)
Heart failure	4.12 (2.74 to 6.19)	3.48 (2.26 to 5.37)	3.72 (2.46 to 5.64)	3.27 (2.09 to 5.09)	3.49 (2.30 to 5.29)
Atrial fibrillation	1.13 (0.67 to 1.91)	1.33 (0.83 to 2.15)	1.39 (0.87 to 2.22)	1.32 (0.82 to 2.13)	1.22 (0.76 to 1.97)
Hypertensive disease	1.27 (1.19 to 1.37)	1.28 (1.20 to 1.37)	1.21 (1.13 to 1.30)	1.13 (1.06 to 1.22)	1.27 (1.19 to 1.35)
Deep vein thrombosis	2.07 (1.55 to 2.76)	2.24 (1.70 to 2.93)	1.95 (1.46 to 2.61)	1.82 (1.35 to 2.47)	1.93 (1.45 to 2.56)

*HR* Hazard ratio, *CI* Confidence interval, *ADHD* Attention-deficit/hyperactivity disorders, *ASD* Autism spectrum disorders, *CVD* Cardiovascular disease

^a^Adjusted for sex, calendar year, parity, maternal age, maternal education, maternal country of origin, maternal psychiatric disorders, and cardiovascular disorders before the childbirth

^b^Including intracranial tumors, head trauma, and intracranial infection

### Sensitivity analyses

Stratification by preterm birth and maternal psychiatric disorders did not indicate any significant differences in the studied associations (product interaction term for preterm birth or maternal history of psychiatric disorders and intellectual disability: *p* = 0.14 and *p* = 0.34, respectively) (Additional file 1: Tables S4-S5). Similar associations were observed in the analyses when restricted the cohort to offspring born after 1995 (Additional file 1: Table S6). The results were in line with those in the main analyses when we included individuals diagnosed with chromosomal abnormalities or congenital heart disease together (Additional file 1: Table S7) or separately (Additional file 1: Table S8-S9).

## Discussion

We observed that individuals who received a diagnosis of intellectual disability had a 24% increased overall risk of early-onset CVD from childhood to early adulthood. In particular, the risks were significantly elevated for the most common specific types of CVD, including cerebrovascular disease (150% increased risk), stroke (120% increased risk), heart failure (256% increased risk), and deep vein thrombosis (110% increased risk). The strongest associations were observed for individuals diagnosed with severe/profound intellectual disability. Excluding neurodevelopmental comorbidity only slightly attenuated the overall risk for CVD.

### Comparison with other studies

To our knowledge, this is the largest study to examine intellectual disability in relation to CVD risk from childhood into adulthood. Our findings are in line with previously reported associations between intellectual disability and hypertension [34], coronary heart disease [15], and heart failure [16]. A few studies showed no significant associations between intellectual disability and CVD [35–38]. For example, a cross-sectional study of 33,122 individuals aged ≥ 18 years with intellectual disability in the USA reported that the prevalence of hypertension in individuals with intellectual disability is similar to that in the general population [35]. Another cross-sectional study of 258 randomly selected adult clients with intellectual disability replicated this finding in the general Dutch population [36]. A retrospective descriptive study of 1333 individuals (510 persons with intellectual disability and 823 general practice patients) aged over 50 years in the Netherlands found that the individuals with intellectual disability were associated with a 50% increased risk of atherosclerotic cardiovascular disease including myocardial infarctions and cerebrovascular diseases, but not statistically significant (relative risk, 1.5; 95% CI, 0.9–2.4) [37]. A cohort study of 790 participants reported that the incidence of cardiovascular disease in people with intellectual disability is similar to that in the general population [38]. These discrepant findings may be due to several factors. The null findings may potentially be due to survivor bias. People with intellectual disability have been recognized as having shortened life expectancy [39]. In these studies, individuals with intellectual disability aged 50 or above may be relatively healthier [36, 37]. On the other hand, the null findings could be due to selection bias. For example, the US study was conducted on individuals with intellectual disability who participated in Special Olympics [35]. The prior null findings may also be influenced by the smaller sample sizes that failed to reach statistical significance [38].

The present study extends prior evidence by comprehensively assessing overall and specific CVD in a large population-based cohort using clinically ascertained diagnoses. The findings showed that the CVD risk is higher in people with intellectual disability compared to the general population and is unsurprising, considering that people with intellectual disability are more likely to have the major risk factors associated with CVD [40, 41]. People with intellectual disability experience higher rates of obesity, diabetes, hyperglycemia, and hyperlipidemia than people without intellectual disability [14, 42]. A meta-analysis consisting of 36,345 participants showed that adolescents with intellectual disability are at respectively 1.54 and 1.80 times higher risk of overweight and obesity than the typically developing adolescents [43]. Individuals with intellectual disability are more likely to lead a sedentary lifestyle with limited physical activity and lack awareness of the negative health impacts of certain risk factors, which could also contribute to the elevated risk of CVD [44, 45]. Furthermore, previous studies have highlighted some issues that are more frequently experienced by people with intellectual disability, such as social exclusion, lower income, and limited access to healthcare and leisure facilities [46, 47], which could predispose them at additional risk of developing CVD. While the risk of overall CVD was elevated both in men and women with intellectual disability, there existed sex differences for some CVD subtypes. We observed that women with intellectual disability were at higher increased risks for ischemic heart disease, heart failure, and deep vein thrombosis. Previous studies that focused on mortality had also found that women with intellectual disability had higher CVD mortality compared with their male counterparts [48, 49]. The mechanisms behind these differences are still unknown.

Clarification of the relative magnitude of the CVD risks for individuals with different levels of intellectual disability severity could assist to formulate the targeting intervention strategies. Yet, no previous study has investigated the CVD risk across the severity of intellectual disability. Previous studies had reported that the risk of premature mortality attributed to somatic conditions increased with the severity of intellectual disability [50]. We found this to be true for CVD. That is, the risk of overall CVD increased with the severity of intellectual disability, and similar patterns were observed for most specific CVD subtypes.

Individuals with intellectual disability have high rates of neurodevelopmental comorbidities [18]. The most prevalent comorbid conditions within those with intellectual disability were ADHD and ASD, which are risk factors for CVD [51, 52]. Our results showed that the risks for overall and specific types of CVD were only slightly attenuated and remained significant after excluding those with different comorbidities, such as ADHD, ASD, and other neurologic disorders.

### Strengths and limitations

This study has several strengths and limitations. First, we investigated a large cohort from Denmark, which include more than 2 million participants. This large cohort with high-quality registries provided sufficient statistical power to examine a comprehensive set of CVD problems. Second, information on intellectual disability was independent of the CVD and free from recall bias. Third, in the Danish registers, the validity of intellectual disability diagnosis has been proven to be high [53]. Our study also had some limitations. First, individuals with intellectual disability were extracted from the patient registry. The prevalence of intellectual disability in our study is in line with previous studies using hospital-based diagnoses extracted from the Danish registry [29, 54]. Nevertheless, no access to intellectual disability diagnosis from primary care, and inclusion of outpatient diagnoses in the DNPR only from 1995 onwards, might potentially have led to the under-detection of intellectual disability cases. Therefore, we could not rule out the possibility of misclassification of intellectual disability. The misclassification of exposure was most likely to be non-differential and would attenuate our estimates to the null, but not likely to over-estimate association. Second, a proportion of intellectual disability was classified as unspecified subtype. The classification of unspecified intellectual disability would not influence the main results on the overall association between intellectual disability and subsequent CVD, but it might overestimate or underestimate the association between severity of intellectual disability and risk of CVD. Third, since the study was conducted using the administrative data, the availability of covariates was limited. For instance, detail information regarding physical activity and stressful life was unavailable to assess for potential confounding by these factors. Fourth, our study population was relatively too young for enough CVD events to occur when our analyses focused on CVD in young and early-middle ages. Thus, it is possible that the studied association was underestimated due to the potential under-detection of CVD. It will be important to re-examine the association in cohorts at an older age to provide a more comprehensive picture.

## Conclusion

The findings from these large national registers provide robust evidence that individuals with intellectual disability have a significantly increased risk of CVD, in particular, for cerebrovascular disease, stroke, heart failure, and deep vein thrombosis even after taking into account a number of confounders and excluding relevant psychiatric commodities. The risks increased with the severity of intellectual disability. The results have implications for the clinical management of individuals with intellectual disability and suggest that screening for CVD problems could become part of the clinical routines. From a public health perspective, the results also highlight the importance of potentially CVD surveillance and early intervention strategies to facilitate efficient and effective care among individuals with intellectual disability in primary health care. Furthermore, our study suggests that promoting a healthy lifestyle program may reduce the excess risk of CVD in community settings.

## Supplementary Information

**Additional file 1:** **Table S1. **The description of registers used in the study. **Table S2. **The diagnostic classification of comorbid neurodevelopmental disorders used in Denmark. **Table S3. **The diagnostic classification of cardiovascular disease in Denmark. **Table S4.** The HRs for the associations between ID and overall and type-specific CVD stratification by preterm birth. **Table S5. **The HRs for the associations between ID and overall and type-specific CVD stratification by maternal psychiatric disorders. **Table S6. **The HRs for the associations between ID and overall and type-specific CVD among all individuals born in Denmark between 1995-2016*.* **Table S7. **The HRs for the associations between ID and overall and type-specific CVD among all individuals born in Denmark between 1978-2016 including individuals diagnosed with chromosomal abnormalities or congenital heart diseases. **Table S8. **The HRs for the associations between ID and overall and type-specific CVD among all individuals born in Denmark between 1978-2016 including individuals diagnosed with congenital heart diseases. **Table S9. **The HRs for the associations between ID and overall and type-specific CVD among all individuals born in Denmark between 1978-2016 including individuals diagnosed with chromosomal abnormalities. **Fig. S1.** Overview of the study population.

## Data Availability

Data were based on Danish national registers, and individual-level data cannot be shared. However, summary statistics, in addition to the results provided in the “Results” section and supplementary material, may be provided if requested.

## Abbreviations

CVD: Cardiovascular disorder CVD
HR: Hazard ratio
ICD-8: International Classification of Disease, Eighth Revision
ICD-10: International Classification of Disease, Tenth Revision
DNPR: Danish National Patient Register
DPCRR: Danish Psychiatric Central Research Register
ADHD: Attention-deficit/hyperactivity disorder
ASD: Autism spectrum disorder
