# Supplementary material for: Association of intellectual disability with overall and type-specific cardiovascular diseases: a population-based cohort study in Denmark
Source: BMC Med. 2023 Feb 6;21:41. doi: 10.1186/s12916-023-02747-4 (PMC9903576; doi:10.1186/s12916-023-02747-4)
Supplement: Supplementary file 1 — Additional file 1: Table S1. The description of registers used in the study. Table S2. The diagnostic classification of comorbid neurodevelopmental disorders used in Denmark. Table S3. The diagnostic classification of cardiovascular disease in Denmark. Table S4. The HRs for the associations between ID and overall and type-specific CVD stratification by preterm birth. Table S5. The HRs for the associations between ID and overall and type-specific CVD stratification by maternal psychiatric disorders. Table S6. The HRs for the associations between ID and overall and type-specific CVD among all individuals born in Denmark between 1995-2016. Table S7. The HRs for the associations between ID and overall and type-specific CVD among all individuals born in Denmark between 1978-2016 including individuals diagnosed with chromosomal abnormalities or congenital heart diseases. Table S8. The HRs for the associations between ID and overall and type-specific CVD among all individuals born in Denmark between 1978-2016 including individuals diagnosed with congenital heart diseases. Table S9. The HRs for the associations between ID and overall and type-specific CVD among all individuals born in Denmark between 1978-2016 including individuals diagnosed with chromosomal abnormalities. Fig. S1. Overview of the study population. [file 12916_2023_2747_MOESM1_ESM.docx]

Additional file 1: Table S1 The description of registers used in the study

| Data source | Information | |
| --- | --- | --- |
| National Patient Register | | Information for all citizens on hospital discharge diagnoses since 1977, and outpatient diagnoses are included from 1995 |
| Medical Birth Register | | Information on gestational age, birth weight, Apgar score at 5 minutes, date of birth, sex, and singleton status since, and maternal smoking during pregnancy since 2004 |
| Danish Civil Registration System | | Information on individual personal identification number, place of birth, marital status, and vital statistics since 1981 |
| Integrated database for labour  market research | | Information on personal labour market affiliation and education establishments since 1981 |
| Psychiatric central research register | | Information of patients treated at psychiatric departments since 1970 |

Additional file 1: Table S2 The diagnostic classification of comorbid neurodevelopmental disorders used in Denmark

| Diseases | ICD codes |
| --- | --- |
| Attention-deficit/hyperactivity disorder | *ICD-8: 308* and *ICD-10*: F90.0, F98.8 |
| Autism spectrum disorder | *ICD-8:* 299 and *ICD-10*: F84 |
| Epilepsy | *ICD-8*: 345 excluding 345.29 and *ICD-10*: G40.0 |
| Cerebral palsy | *ICD-8*: 343.99, 344.99 and *ICD--10*: G80 |
| Intracranial tumors | *ICD-8*: 191, 225 and *ICD-10*: C70-C71, D32-D33 |
| Severe head trauma | *ICD-8*: 851, 854 and *ICD-10*: S06.1-S06.9 |
| Intracranial infection | *ICD-8*: 320, 323 and *ICD-10*: G00-G09 |

ICD: International Statistical Classification of Diseases and Related Health Problems.

Additional file 1: Table S3 The diagnostic classification of cardiovascular disease in Denmark

|  | ICD codes |
| --- | --- |
| Ischemic heart disease | *ICD-8*: 410-414 and *ICD-10*: I20-I25 |
| Cerebrovascular disease | *ICD-8*: 430-438 and *ICD-10*: I60-I69 |
| Stroke | *ICD-8*: 430-436 and *ICD-10*: I61-I64 |
| Heart failure | *ICD-8*: 427.0, 427.1, 782.4 and *ICD-10*: I110, I130, I132, I50 |
| Atrial fibrillation | *ICD-8*: 427.93, 427.94 and *ICD-10*: I48 |
| Hypertensive disease | *ICD-8*: 400-404 and *ICD-10*: I10-I15 |
| Deep vein thrombosis | *ICD-8*: 451.00 and *ICD-10*: I80.1-I80.3 |

ICD: International Statistical Classification of Diseases and Related Health Problems.

Additional file 1: Table S4 The HRs for the associations between ID and overall and type-specific CVD stratification by preterm birth

|  | Preterm birth | |
| --- | --- | --- |
|  | No | Yes |
|  | HR (95% CI) | HR (95% CI) |
| Overall CVD | 1.20 (1.10 to 1.31) | 1.46 (1.19 to 1.81) |
| Ischemic heart diseases | 1.08 (0.71 to 1.67) | 2.18 (1.02 to 4.68) |
| Cerebrovascular diseases | 2.33 (1.83 to 2.96) | 3.44 (2.03 to 5.82) |
| Stroke | 2.05 (1.52 to 2.75) | 2.94 (1.55 to 5.58) |
| Heart failure | 3.65 (2.34 to 5.71) | 2.17 (0.68 to 6.95) |
| Atrial fibrillation | 1.26 (0.76 to 2.10) | 0.55 (0.08 to 3.96) |
| Hypertensive disease | 1.26 (1.17 to 1.35) | 1.15 (0.95 to 1.39) |
| Deep vein thrombosis | 2.00 (1.48 to 2.70) | 2.12 (0.99 to 4.53) |

Adjusted for sex, calendar year, parity, maternal age, maternal education, maternal country of origin, maternal psychiatric disorders, and cardiovascular disorders before childbirth; HR: hazard ratio; CI: confidence interval; ID indicates intellectual disability; CVD indicates cardiovascular diseases.

Additional file 1: Table S5 The HRs for the associations between ID and overall and type-specific CVD stratification by maternal psychiatric disorders

|  | Maternal psychiatric disorders | |
| --- | --- | --- |
|  | No | Yes |
|  | HR (95% CI) | HR (95% CI) |
| Overall CVD | 1.22 (1.13 to 1.33) | 1.38 (1.09 to 1.75) |
| Ischemic heart diseases | 1.16 (0.72 to 1.87) | 1.27 (0.72 to 2.25) |
| Cerebrovascular diseases | 3.05 (2.39 to 3.90) | 1.42 (0.91 to 2.21) |
| Stroke | 2.79 (2.08 to 3.75) | 1.09 (0.62 to 1.93) |
| Heart failure | 3.92 (2.42 to 6.35) | 2.68 (1.26 to 5.70) |
| Atrial fibrillation | 1.07 (0.57 to 2.00) | 1.52 (0.75 to 3.06) |
| Hypertensive disease | 1.32 (1.24 to 1.41) | 1.27 (1.20 to 1.36) |
| Deep vein thrombosis | 2.08 (1.48 to 2.92) | 1.95 (1.25 to 3.04) |

Adjusted for sex, calendar year, parity, maternal age, maternal education, maternal country of origin, and cardiovascular disorders before childbirth; HR: hazard ratio; CI: confidence interval; ID indicates intellectual disability; CVD indicates cardiovascular diseases.

Additional file 1: Table S6 The HRs for the associations between ID and overall and type-specific CVD among all individuals born in Denmark between 1995-2016

|  | Rate per 1000 person-years | | HR (95% CI) | |
| --- | --- | --- | --- | --- |
|  | Individuals without ID | Individuals with ID | Model 1 | Model 1 |
| Overall CVD | 1.14 | 1.43 | 1.21 (1.00 to 1.45) | 1.21 (1.00 to 1.45) |
| Type-specific CVD |  |  |  |  |
| Ischemic heart diseases | 0.01 | 0.01 | 0.70 (0.10 to 4.99) | 0.60 (0.08 to 4.30) |
| Cerebrovascular diseases | 0.07 | 0.14 | 2.33 (1.32 to 4.12) | 2.23 (1.26 to 3.96) |
| Stroke | 0.05 | 0.08 | 1.83 (0.87 to 3.86) | 1.76 (0.83 to 3.71) |
| Heart failure | 0.01 | 0.01 | 1.05 (1.15 to 7.51) | 1.09 (0.15 to 7.79) |
| Atrial fibrillation | 0.01 | 0.04 | 3.11 (0.99 to 9.77) | 3.02 (0.96 to 9.54) |
| Hypertensive disease | 3.44 | 2.43 | 0.76 (0.66 to 0.88) | 0.73 (0.63 to 0.84) |
| Deep vein thrombosis | 0.02 | 0.02 | 1.23 (0.31 to 4.95) | 1.11 (0.27 to 4.47) |

Model 1 adjusted for sex and calendar year; Model 2 additionally adjusted for parity, maternal age, maternal education, maternal country of origin, maternal psychiatric disorders and cardiovascular disorders before childbirth; HR: hazard ratio; CI: confidence interval; ID indicates intellectual disability; CVD indicates cardiovascular diseases.

Additional file 1: Table S7 The HRs for the associations between intellectual disability and overall and type-specific CVD among all individuals born in Denmark between 1978-2016 including individuals diagnosed with chromosomal abnormalities or congenital heart diseases

|  | HR (95% CI) | |
| --- | --- | --- |
|  | Model 1 | Model 2 |
| Overall CVD | 1.44 (1.35 to 1.54) | 1.37 (1.28 to 1.47) |
| Ischemic heart diseases | 1.65 (1.22 to 2.23) | 1.40 (1.02 to 1.90) |
| Cerebrovascular diseases | 2.57 (2.12 to 3.13) | 2.45 (2.01 to 2.99) |
| Stroke | 2.33 (1.85 to 2.95) | 2.21 (1.74 to 2.81) |
| Heart failure | 3.31 (2.47 to 4.45) | 3.20 (2.37 to 4.34) |
| Atrial fibrillation | 1.68 (1.17 to 2.41) | 1.53 (1.04 to 2.23) |
| Hypertensive disease | 1.44 (1.36 to 1.52) | 1.42 (1.34 to 1.50) |
| Deep vein thrombosis | 2.31 (1.82 to 2.94) | 2.14 (1.67 to 2.74) |

Adjusted for sex, calendar year, parity, maternal age, maternal education, maternal country of origin, and cardiovascular disorders before childbirth; HR: hazard ratio; CI: confidence interval; ID indicates intellectual disability; CVD indicates cardiovascular diseases.

Additional file 1: Table S8 The HRs for the associations between intellectual disability and overall and type-specific CVD among all individuals born in Denmark between 1978-2016 including individuals diagnosed congenital heart diseases

|  | HR (95% CI) | |
| --- | --- | --- |
|  | Model 1 | Model 2 |
| Overall CVD | 1.35 (1.25 to 1.45) | 1.28 (1.18 to 1.37) |
| Ischemic heart diseases | 1.64 (1.20 to 2.25) | 1.37 (0.99 to 1.89) |
| Cerebrovascular diseases | 2.62 (2.14 to 3.21) | 2.48 (2.02 to 3.05) |
| Stroke | 2.32 (1.81 to 2.97) | 2.19 (1.70 to 2.81) |
| Heart failure | 2.80 (1.97 to 3.98) | 2.74 (1.92 to 3.92) |
| Atrial fibrillation | 1.54 (1.04 to 2.29) | 1.37 (0.90 to 2.08) |
| Hypertensive disease | 1.35 (1.27 to 1.44) | 1.33 (1.25 to 1.41) |
| Deep vein thrombosis | 2.29 (1.77 to 2.95) | 2.10 (1.61 to 2.73) |

Adjusted for sex, calendar year, parity, maternal age, maternal education, maternal country of origin, and cardiovascular disorders before childbirth; HR: hazard ratio; CI: confidence interval; ID indicates intellectual disability; CVD indicates cardiovascular diseases.

Additional file 1: Table S9 The HRs for the associations between intellectual disability and overall and type-specific CVD among all individuals born in Denmark between 1978-2016 including individuals diagnosed with chromosomal abnormalities

|  | HR (95% CI) | |
| --- | --- | --- |
|  | Model 1 | Model 2 |
| Overall CVD | 1.35 (1.25 to 1.45) | 1.29 (1.19 to 1.39) |
| Ischemic heart diseases | 1.45 (1.03 to 2.04) | 1.20 (0.84 to 1.71) |
| Cerebrovascular diseases | 2.59 (2.11 to 3.18) | 2.48 (2.02 to 3.06) |
| Stroke | 2.31 (1.80 to 2.97) | 2.18 (1.69 to 2.81) |
| Heart failure | 3.88 (2.66 to 5.64) | 3.55 (2.40 to 5.25) |
| Atrial fibrillation | 1.30 (0.84 to 2.02) | 1.24 (0.79 to 1.95) |
| Hypertensive disease | 1.37 (1.29 to 1.46) | 1.35 (1.27 to 1.43) |
| Deep vein thrombosis | 2.27 (1.76 to 2.92) | 2.09 (1.61 to 2.71) |

Adjusted for sex, calendar year, parity, maternal age, maternal education, maternal country of origin, and cardiovascular disorders before childbirth; HR: hazard ratio; CI: confidence interval; ID indicates intellectual disability; CVD indicates cardiovascular diseases.


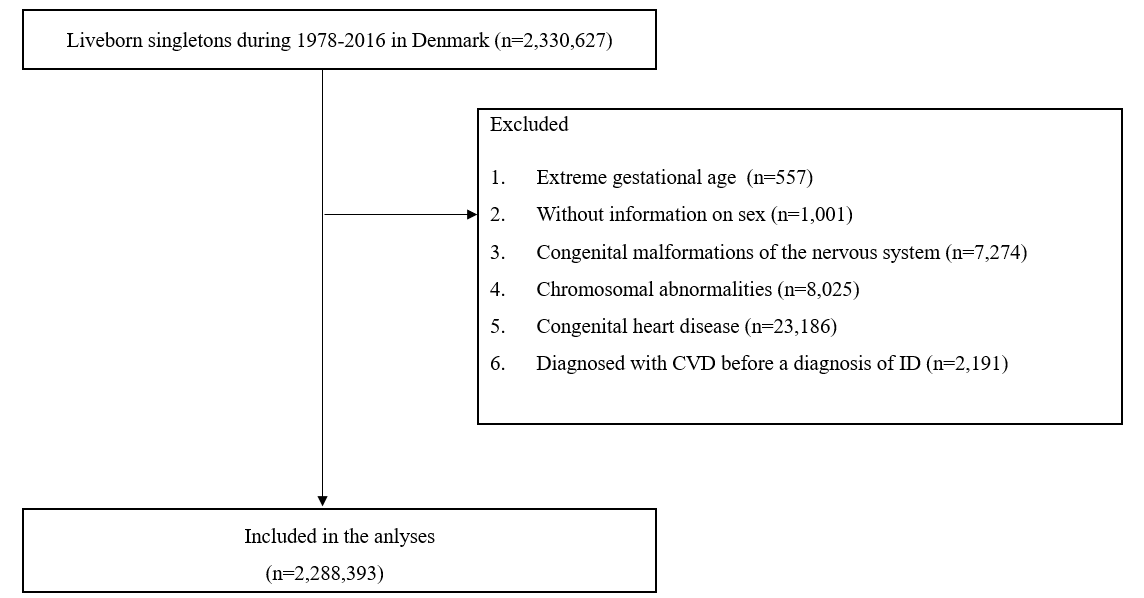


Additional file 1: Figure S1 Overview of the study population
